# Hiking, indoor biking, and body liking: a cross-sectional study examining the link between physical activity arenas and adults’ body appreciation

**DOI:** 10.1186/s40337-022-00705-8

**Published:** 2022-11-24

**Authors:** Christine Sundgot-Borgen, Lise Katrine Jepsen Trangsrud, Tobias Otterbring, Solfrid Bratland-Sanda

**Affiliations:** 1 Regional Department for Eating Disorders, Division of Mental Health and Addiction, Oslo University Hospital, Postbox 4956, 0424 Nydalen, Oslo Norway; 2 Department of Health, Social and Welfare Studies, University of South-Eastern Norway, P.O. Box 235, 3603 Kongsberg, Norway; 3 Department of Management, University of Agder, Postboks 422, 4604 Kristiansand, Norway; 4 Department of Sports, Physical Education and Outdoor Studies, University of South-Eastern Norway, P.O. Box 235, 3603 Kongsberg, Norway

**Keywords:** Body image, Body appreciation, Physical activity, Connectedness with nature, Self-compassion

## Abstract

**Background:**

Body appreciation might serve as a protective factor for developing eating disorders and is associated with participation in physical activity. Less is known about whether various arenas for physical activity may be linked to body appreciation. Therefore, the current study sought to (1) identify potential associations between physical activity level and arenas for physical activity, connectedness with nature, self-compassion, and body appreciation in adults, and (2) explore physical activity level and arenas, connectedness with nature, and self-compassion as explanatory factors for body appreciation.

**Methods:**

A total of 360 adult Norwegian inhabitants (75% women, mean (SD) age 42.58 (12.30) yrs) responded to an online questionnaire. Instruments included the Body Appreciation Scale, the Connectedness with Nature Scale, and the Self-Compassion Scale.

**Results:**

The percentage of participants who engaged in various physical activity arenas were 98.5% for nature, 57.5% for fitness centers, and 43.0% for organized sports. Small, positive associations were found between body appreciation and the frequency of using fitness centers and nature as physical activity arenas. Self-compassion, connectedness with nature, and frequency of using fitness centers and nature as physical activity arenas explained 39% of the variance in body appreciation.

**Discussion:**

The importance of both fitness centers and nature as arenas for physical activity to explain body appreciation was surprising and might reflect different use of fitness centers among adults compared to younger age groups.

**Conclusion:**

Physical activity at fitness centers and in nature were positively associated with body appreciation in adults. Self-compassion, connectedness with nature, and using fitness centers and nature as arenas for physical activity, were found to explain variation in body appreciation in adults.

## Plain English summary

Physical activity helps us feel good about ourselves and appreciate our bodies. However, less is known about the extent to which different arenas for physical activity are related to body appreciation. Therefore, 360 adults from Norway completed a survey with questions about their physical activity level, use of nature, fitness centers, and organized sports as arenas for physical activity, and measures linked to connectedness with nature, self-compassion, and body appreciation. We found an association between body appreciation and performing physical activity at fitness centers and in nature. Physical activity at these two arenas together with self-compassion and connectedness with nature explained body appreciation in these adults. Future studies should focus on the adolescent population, where the level of body appreciation tend to be lower and where organized sports, in addition to fitness centers, constitute an even more commonly used physical activity arena.

## Background

Several studies have highlighted that a large proportion of women and men report a difficult relationship with their own bodies [1, 2]. Body dissatisfaction is identified as one of the cardinal predictors of eating disorders [3–5], and has been associated with risk of depressive symptoms [6] and obesity [7]. By contrast, a positive body image may serve as a protective factor for developing eating disorders [8, 9], and has been found to be positively associated with good mental health and healthy lifestyle choices, including engagement in physical activity [10, 11]. Components of a positive body image relate to appreciation and acceptance of one’s body, regardless of its appearance, as well as displaying gratitude towards the functionality and health of the body [12]. Importantly, the identification of factors that protect and promote the development of a positive body image, including various contextual factors such as arenas for physical activities, has been called for in recent research [12].

Participation in physical activity, defined as bodily movements induced by skeletal muscles leading to an increased energy expenditure [13], holds several mental health benefits [14], including a positive body image [15]. However, this positive association might be influenced by the arena in which the physical activities take place. As such, studies have found that fitness centers, which represent the leading indoor physical activity arena for adults, are associated with experienced body appearance pressure among young adults [16, 17]. This experienced pressure might negatively influence the otherwise positive relationship between physical activity and body image in this context.

As a contrast to the above, research shows that physical activity in natural, outdoor environments, such as urban green areas, parks, and forests, often referred to as green exercise, contribute more to mental health and emotional wellbeing compared to physical activity in built, indoor, or maintained environments [18, 19]. Specifically, physical activity in blue or larger coherent natural areas, such as forest or wildlands, have been found to hold restorative benefits compared to physical activity in other outdoor environments, and suggested mechanisms for these benefits are the lower levels of environmental and social stressors such as noise, traffic, or other people [18].

The health-related benefits of exposure to, and engagement with nature are well documented [20–24]. Studies from across the world report enhanced body appreciation following nature-contact and nature-based activities [25–27]. For instance, in a systematic review of outdoor activities as part of eating disorder recovery, participation in outdoor activities was perceived to shift focus from physical appearance to physical ability, while simultaneously challenging the prevailing body image and enabling individuals to re-evaluate gendered roles and stereotypes identified in society [25]. However, the outcome of existing studies is frequently based on a mix of factors related to the actual activity, the interpersonal relationships in nature, and the surroundings for the activity that the natural environments represent [28, 29]. Additionally, as engagement with nature may consist of both contemplative activities and their more physically demanding counterparts, risks have been raised with respect to confounding the effects of spending time in natural environments with the effects of physical activity. As such, there is a need to increase knowledge about the environmental settings that provide synergies between physical activity and mental health [18]. While experimental studies have been important in demonstrating health-related benefits of nature contact, several scholars have called for more research that investigates whether the same effects as those found in controlled experiments can be identified in more ecologically valid settings [18, 30]. Knowledge related to possible associations between arenas for physical activity and a positive body image is also important for recommendations and motivation towards physical activity in the general population.

As for other body image constructs, young adult males report higher levels of body appreciation relative to young adult females [31]. Interestingly, this gender difference might be moderated by age [31]. Studies have found that in contrast to body dissatisfaction, an increased age is associated with a more positive body image especially in females [32]. Hence, studies should take gender and age into consideration when investigating factors that may be associated with body appreciation.

Notably, one study found that self-compassion mediated the positive effects from nature exposure on body appreciation [33], thus indicating that self-compassion may act as a psychological mechanism that explains why nature exposure is associated with increased body appreciation. Accordingly, self-compassion should also be included in models aimed at examining people’s body image and related phenomena.

The current study sought to (1) identify potential associations between physical activity level and arenas for physical activity, connectedness with nature, self-compassion, and body appreciation, and (2) investigate potential explanatory factors for body appreciation such as (a) physical activity level, (b) the frequency with which people engage in physical activity in organized sports, fitness centers, and nature, and (c) their connectedness with nature as well as their self-compassion.

## Method

### Study design

The study was part of a larger research project that aimed to examine people’s body image in nature across several countries and continents [34]. For this current study, the data were collected solely in Norway.

### Participant recruitment and data collection

The sample consisted of 360 Norwegian citizens aged 18 years or older, of all genders. With an alpha level of *α* = 0.05, the sample size has a statistical power of approximately 0.80 to detect small-to-moderate effect sizes corresponding to *r* = .15 [35]. Inclusion criteria were adequate language skills to complete the survey in Norwegian. Participants were recruited through social media (Facebook, Instagram, Twitter) as well as university networks. They were informed that the study aimed to investigate the association between nature exposure and psychological well-being, and that they were asked to respond to an electronic questionnaire that included questions related to nature exposure, connectedness to nature, mental health, body image, and physical activity, and that the estimated response time was approximately 15 min. All respondents provided a written consent form before they got access to the questionnaire. No compensation was given for taking part in the study.

### Ethics

The study was approved by the Norwegian Data Protection Service (ID 833,522). Participants were informed that participation was voluntary, and that their identity in no way could be connected to their responses, as they replied to the survey through a link without response identification. No personal identification questions were asked.

### Measurements

#### Demographics

Participants self-reported age, gender (male, female, other), height (meter), and weight (kg). Body mass index (BMI) was calculated from self-reported height and weight (kg/m^2^). Participants were also asked whether they belonged to the ethnic minority or majority in Norway, if they lived in urban or rural areas, and indicated their educational status.

#### Body appreciation scale, version 2 (BAS-2)

BAS-2 measures body appreciation through 10 items, where participants respond to a Likert scale ranging from 1 (*never*) to 5 (*always*), with a higher average score indicating a higher level of body appreciation [36]. In the present study, Cronbach’s alpha (α) was 0.92 and 0.94 in male and females, respectively, which is similar to what has been found among Norwegian male and female students in prior research [17].

#### Physical activity

The respondents were asked to report how many hours during a regular week they were physically active to a level where they felt increased body temperature and shortness of breath. They were also asked about the frequency of physical activity at the arenas “organized sport”, “fitness center”, and “in nature”, where the latter was responded to on a Likert sale ranging from 1 (*Never*) to 7 (*Every day*).

#### Connectedness with nature scale

To measure an individual’s trait levels of feeling emotionally connected to the natural world, we used the Connectedness with Nature Scale. Participants replied to the 14 items using a 5-point Likert scale ranging from 1 (*strongly disagree*) to 5 (*strongly agree*). Negative items were reversed (items 4, 12, and 14), and a mean score was calculated, where higher means indicate higher levels of connectedness with nature. Cronbach’s alpha was 0.90 (0.88 for males and females, respectively), with these reliability coefficients being slightly higher than for the original American male and female community and student sample (0.84) [37].

#### Self-compassion scale, short-form

Self-compassion was measured by the Self-Compassion Scale-Short Form [38]. Participants replied to the 12 items through a Likert scale anchored at 1 (*almost never*) and 5 (*almost always*), with total (sum) scores ranging from 12 to 60. All negatively worded items were reversed (items 1, 4, 8, 9, 11, and 12). Cronbach’s alpha was 0.88 and 0.94 in males and females, respectively, again slightly higher than for the original English adult sample (0.86) [38].

### Statistics

Data were analyzed with IBM SPSS version 26. After visually evaluating the data for normality, between-group differences were analyzed using Student’s independent samples *t*-test for parametric data and Pearson’s chi-square test for categorical data. Hierarchical multiple regression was used to investigate partial correlations between the dependent variable (body appreciation) and the independent variables (frequency of physical activity, arena use, and mental health variables), while controlling for age and gender. A three step Hierarchical multiple linear regression model was used to investigate explanatory factors for the variance in body appreciation, controlling for age and gender. Effect sizes are presented as Hedges’ g (*g*) and Phi-coefficient (φ) for parametric and non-parametric data, respectively. Standardized Beta Coefficients (ß) describes the regression effect sizes [39].

## Results

### Demographics

The average male and female participant was above 40 years old, classified as overweight, represented the ethnic majority in Norway, were highly educated, and reported to live in rural areas. Except for a higher self-compassion score among males, no gender differences were found in mental health variables (Table 1).

**Table 1:** Participant descriptions and differences between males and females

	Males (N = 88)	Females (N = 277)			
	M (SD)	M (SD)	Mean diff. [CI 95%]	*p*	*g*
Age	45.00 (13.90)	40.17 (10.70)	− 4.83 [− 8.14, − 1.51]	**0.005**	0.43
BMI kg/m^2^	26.29 (4.16)	25.03 (4.81)	− 1.26 [− 2.42, − 0.10]	**0.033**	0.27
Ethnical minority	87 (99%)^a^	262 (94%)^b^	5%	0.266	
Urban versus rural citizens	28 (35%)	108 (39%)	4%	0.726	
Higher education	63 (80%)^a^	246 (91%)^c^	2%	**0.024**	0.15*
PA h/week	6.04 (5.44)	6.67 (22.00)	0.63 [− 4.22, 5.49]	0.797	
BAS-2	3.84 (0.67)	3.68 (0.71)	− 0.16 [− 0.34, 0.01]	0.068	
CNS	3.45 (0.81)	3.55 (0.69)	0.10 [− 0.09, 0.30]	0.303	
SCS	41.30 (8.51)	39.04 (9.22)	− 2.26 [− 4.51, − 0.01]	**0.049**	0.25

Table shows gender-specific means (SDs) and mean differences between male and female participants (*Mean diff)*; *Age* Years of age; *BMI* Body mass index (kg/m^2^); *PA h/week* Amount of hours reported to be physically active during one week; *BAS-2* Body appreciation scale-2; *CNS* Connectedness with nature scale; *CI* Confidence interval. A *p*-value of ≤ 0.05 is set as statistically significant when comparing groups (statistically significant *p* values are presented in bold). *g*: Hedges’ g and *Phi-coefficient is reported where there is a significant group difference. ^a^N = 79 ^b^N = 272 ^c^N = 271. Urban citizens = includes participants responding that they live in the capital city or a city. Rural citizens = represent participant from a provincial town or rural areas. Higher education = Still in full-time education, undergraduate degree, or postgraduate degree

### Use of physical activity arenas

As presented in Table 2, almost all male and female participants used nature as an arena for physical activity, while more than 50% of male and females used fitness centers, and a lower percentage used organized sports, with no gender differences.

**Table 2:** Number and percentage of participants engaged in each physical activity arena

	Males (N = 81)	Females (N = 277)	*p*
	N (%)	N (%)	
PA in nature	79 (98)	275 (99)	0.414
PA at fitness center	47 (58)	159 (57)	0.259
PA in organized sports	38 (47)	107 (39)	0.395

Table shows number (N) and percentage (%) of all participants engaged in physical activity (PA) in different arenas, and differences between males and females. Participants were able to report being member in more than one PA arena. A *p*-value of ≤ 0.05 is set as statistically significant when comparing two groups

### Associations between body appreciation, physical activity arenas and mental health

Partial correlational analyses revealed that participants who reported more hours of physical activity per week and a higher frequency of use of fitness centers and nature as arenas for physical activity, also reported higher levels of body appreciation. When controlling for age and gender (male = 1, female = 0), the strength of the correlation between body appreciation and hours in physical activity and the use of fitness centers increased, whereas the strength decreased between body appreciation and the use of nature as a physical activity arena, connectedness with nature, and self-compassion. (Table 3).

**Table 3:** Partial correlation between body appreciation, frequency of physical activity arena used, physical activity level, and mental health variables

	BAS-2
PA in organized sports	0.07
PA at fitness center	0.14**
PA in nature	0.21**
PA h/week	0.14**
CNS	0.24**
SCS	0.55**

Partial correlations after controlling for age and gender. *BAS-2* Body appreciation scale-2; *CNS* Connectedness with nature scale; *SCS* Self-compassion scale short-form. *Correlations are set as statistically significant at a *p*-value of ≤ 0.05

Hierarchical multiple regression was used to investigate explanatory factors for the variance in body appreciation. Preliminary analyses were conducted to ensure no violation of the assumptions of normality, linearity, multicollinearity, and homoscedasticity. Age, gender, self-compassion, connectedness with nature, physical activity level, and frequency of use of fitness centers and nature were included in the model. Frequency of using organized sports was excluded because this variable did not correlate with body appreciation (Table 3).

Step one, including age and gender, explained 6.3% of the variance in body appreciation, *F*(2, 355) = 11.988 *p* < .001. After entering self-compassion and connectedness with nature in step 2, the total variance explained by the model was 36.5%, *F*(4, 353) = 50.76 *p* < .001. The two variables explained an additional 30.2% of the variance in body appreciation after controlling for age and gender. In step 3, physical activity level and frequency of using fitness centers and nature as physical activity arenas explained an additional 2.6% of the variance after controlling for self-compassion and connectedness with nature. The overall model now explained 39.1%, *F*(7, 350) = 32.08 *p* < .001 of the variance in body appreciation. As seen in Table 4, the strongest predictor based on standardized ß was self-compassion (ß = 0.51), followed by connectedness with nature (ß = 0.12), and frequency of using fitness centers (ß = 0.11) and nature (ß = 0.10) as physical activity arenas (Table 4).

**Table 4:** Hierarchical Regression Analysis Predicting body appreciation

		B	Std. E	ß	*p*	R^2^
Step 1	(Constant)	3.11	0.13		**< 0.001**	0.06*
	Age	0.01	0.00	0.24	**< 0.001**	
	Gender	0.09	0.09	0.06	0.285	
Step 2	(Constant)	1.47	0.19		**< 0.001**	0.30*
	Age	0.00	0.00	0.06	0.206	
	Gender	0.07	0.07	0.04	0.367	
	SCS	0.04	0.00	0.53	**< 0.001**	
	CNS	0.13	0.04	0.13	**0.004**	
Step 3	(Constant)	1.19	0.20		**< 0.001**	0.03*
	Age	0.00	0.00	0.06	0.224	
	Gender	0.08	0.07	0.05	0.257	
	SCS	0.04	0.00	0.51	**< 0.001**	
	CNS	0.12	0.05	0.12	**0.007**	
	PA h/week	0.00	0.01	0.02	0.622	
	Fitness centers	0.04	0.02	0.11	**0.010**	
	Nature	0.05	0.02	0.10	**0.049**	

*B* Unstandardized beta coefficient; *Std. E* Standard error; *ß* Standardized beta coefficients; *R*^2^ R square change; *BAS-2* Body appreciation scale-2; *CNS* Connectedness with nature scale; *SCS* Self-compassion scale, short-form. Fitness center = frequency in which fitness center is used to be physically active. Nature = frequency in which the nature is used to be physically active. *Correlations are set as statistically significant at a *p*-value of ≤ 0.05. Statistically significant *p* values are presented in bold

## Discussion

This study aimed to identify associations between physical activity level and arenas, connectedness with nature, self-compassion, and body appreciation, and to explore to what extent these factors would play a role in explaining variation in body appreciation among a community sample of Norwegian participants. As the main findings, we found that although self-compassion explained most of the variance in participants’ body appreciation, both fitness centers and nature as arenas for physical activity explained a meaningful portion of the variance in body appreciation, even after controlling for connectedness with nature and self-compassion.

Surprisingly, there was no important significant difference between frequency of using fitness centers or nature as physical activity arena and their ability to explain variance in body appreciation when controlling for the other variables in our tested model. Therefore, prior findings of outdoor physical activity arenas as significantly more positive for body appreciation compared to indoor arenas [18, 19] were not reflected in the current work. In fact, our fitness center finding was particularly puzzling considering previous studies, which have found fitness centers to be potentially destructive to members’ and employees’ body image due to the appearance focus and body appearance pressure found in such activity arenas [16, 17]. Importantly, our results did not reflect a negative association between using fitness centers as physical activity arenas and participants’ body appreciation. Two potential explanations for this finding are the age profile of our sample and the sociocultural position of fitness centers in Norway.

First, the mean age of participants was above 40 years. People in this age group may use fitness centers differently compared to younger adults. Indeed, motivation for physical activity changes with age, and although weight- and appearance-related reasons remain important, middle-aged and older adults perceive health and illness prevention as more important reasons for physical activity [40, 41].

Second, the focus of fitness centers in Norway has shifted during the past decades from solely focusing on muscle building and appearance for highly selected gym users, to a public health facility [42]. For middle aged and older adults, the physical activity in fitness centers might therefore contribute to the experience of a strong, enduring, and well-functioning body, which may in turn be positively associated with body appreciation [43], as has been suggested as a consequence of being physically active in natural environments [33].

Interestingly, controlling for age and gender strengthened the positive correlation between frequency of using fitness centers and body appreciation as well as physical activity levels and body appreciation, while this correlation was reduced between connectedness with nature and using nature as a physical activity arena. This could indicate that the correlation between the frequency of using fitness centers and the level of physical activity is stronger among young individuals and males compared to their older and/or female counterparts, while the opposite is true for connectedness with nature, frequency of using nature as arena, and body appreciation. Interestingly, both age and gender failed to explain a meaningful portion of the variance in body appreciation when the other variables were entered into the model and, as such, could not be established as predictors for body appreciation.

Partial correlations showed no association between body appreciation and sports participation, which is surprising considering other related studies suggesting a significant relationship between these factors [44–46]. This difference might relate to the age difference between our sample and samples in previous studies. As we did not collect any data about the levels (e.g., recreational vs. competitive sports) or types of sports that participants engaged in, such variables could serve as fruitful avenues for future research. For example, participation in high-level competitions in weight-sensitive sports such as cross-country skiing and running increases risk of body dissatisfaction and the development of eating disorders [47]. Whether such participation is also negatively associated with body appreciation is yet to be examined.

Findings from the current study demonstrated that self-compassion explained most of the variance in participants’ body appreciation. This echoes findings from a meta-analysis which found that self-compassion moderately (r = .55) correlated with body appreciation [48], and agrees with another study which found that body appreciation at follow-up was predicted by higher levels of self-compassion [49]. In the current study, following self-compassion, with a substantially smaller effect size, connectedness with nature also took part in explaining the variance in participants’ body appreciation. This is in line with previous research highlighting that both exposure to natural environment [50, 51] and a sense of connectedness with nature were associated with higher body appreciation among both males and females [52]. Interestingly, recent research has reported how the specific natural environment matters. Here, wood- and grassland, mountains and particularly blue areas were found significantly associated with higher physical appearance satisfaction, while exposure to urban green-areas was not [27]. In our study, the majority of the participants resided in rural areas. Although it can be argued that nature is relatively accessible also in the more urban areas of Norway, previous research has demonstrated how restorative benefits of nature were most likely experienced in larger coherent areas of nature as well as near blue areas [53]. A preference for qualities found in nature outside of urban contexts, such as silence and calmness, great views, and an experience of being part of nature’s own balance, have been highlighted as important in a shift from physical appearance towards appreciation and acceptance of own body [54, 55]. Our findings show that connectedness with nature prevails the level of, and arena for, physical activity, in terms of association with body appreciation. This is interesting given earlier concerns related to the risk of confounding physical activity and nature-contact when seeking to explain restorative benefits [53], and support the emphasis on the arena for developing healthy relationships with own body.

Nevertheless, it is important to point out that the standardized regression coefficients for both connectedness with nature, and fitness centers and nature as arenas for physical activity, reflect significant but small predictor effects on body appreciation. In addition to self-compassion, unmeasured constructs seem to play an additional role, and are important to discuss and investigate in future research to fully understand how body appreciation can be promoted. In line with our finding on self-compassion’s predictive role, working on one’s self-compassion can improve body appreciation [56]. Other intervention studies have found that engaging in yoga [57], improved focus on functionality [58, 59], and enhanced mindfulness [60], can promote body appreciation.

Except for higher scores on self-compassion among males, no gender differences were found in body appreciation, connectedness with nature, or use of the different physical activity arenas. The lack of gender difference in body appreciation, as reported herein, is in conflict with the majority of studies finding that males report a higher body appreciation compared to females [31]. However, it is important to point out that our male and female sample represented individuals with a higher mean age compared to adolescents and young adults who are more often investigated in previous studies on body appreciation. This is important to take into consideration, as prior studies have found that the gender difference in body appreciation seems to fade off as females increase in age [31, 32]. The age of our sample could thus potentially explain our obtained lack of gender differences.

### Strengths and limitations

The strengths of the current study include a sample of both males and females, as the majority of body image research has focused on females. In addition, the higher mean age compared to many other studies provides novel information on body appreciation and associated factors in a more mature sample. We also used validated and standardized instruments for the assessment of body appreciation, self-compassion, and nature connectedness.

However, the findings are limited by a potential selection bias. Participants were assumed to have a specific interest in nature (due to the survey focus), had high self-reported levels of physical activity, were almost entirely restricted to the ethnic majority in Norway, and were by and large highly educated, with the latter factor often being linked to an increased propensity to engage in outdoor activities [61]. Further, our sample had a skewed ratio of male/female respondents. Moreover, we cannot rule out the potential presence of colliders in our analyses, and our cross-sectional design does not allow us to draw causality conclusions with certainty; but see [62]. Relatedly, our regression model only explained parts of the variance in body appreciation, which means that other unmeasured factors also need to be considered in future scholarly work. Finally, one potential limitation may be related to the translation of the measurement scales from English to Norwegian. Although this was controlled for by back-translating from Norwegian to English, some categories or phrases were kept close to their original wording. This makes it easier to compare the results between different cultural settings but may not capture the Norwegian cultural context in all questions and could potentially lead to misunderstandings.

### Future research and practical implications

Future studies should aim to develop experimental designs to better understand the direction of the observed associations and to determine the causal relationship between nature connectedness, arenas for physical activity, and body appreciation. Future research also needs to examine if the pattern found in this study can be seen in younger adults, including more males and across a broader range of educational levels. Also, larger sample sizes would allow further sub-group analyses, such as testing whether age and gender may moderate certain associations. Additionally, a wider recruitment could reduce the risk of selection bias and improve generalizability. Notwithstanding the study limitations, our findings still indicates that among others, engaging in physical activity within both fitness centers and nature may be an important factor that can influence people’s body appreciation.

## Conclusion

Self-compassion, connectedness with nature, and physical activity in both fitness centers and nature, but not in organized sports, were positively associated with body appreciation in adults. Self-compassion, connectedness with nature, and fitness centers and nature as arenas for physical activity, explained 39% of the variance of body appreciation. These findings have theoretical and practical implications, and are important not least because they run contrary to previous studies regarding the fitness center as an arena for developing body appreciation.

## Data Availability

The datasets used and/or analysed during the current study are available from the corresponding author on reasonable request.

## Abbreviations

BMI: Body mass index
BAS-2: Body appreciation scale-version 2
CNS: Connectedness with nature scale
SCS: Self-compassion scale short-form
PA: Physical activity
